# On Aconite as a Therapeutical Agent

**Published:** 1879-04

**Authors:** James S. Spark


### ON ACONITE AS A THERAPEUTICAL AGENT.

By JAMES S. SPARK.

There are few, if any, of the many drugs which have come into use, or been prominently before the profession, of late years, more deserving of notice, or more useful to the general practitioner, than aconite. But so much has been done by Fleming and others, in investigating its physiological and therapeutical action, and every now and then our attention is called to some new case in which it has been found of service, it is surprising how little it is employed by practitioners throughout the country. There are many inflammatory affections where its effects are literally marvelous, not only from the efficacy, but also from the rapidity of its action, cases in which none of the usual *routine* drugs operate either so unmistakably or so efficiently, if indeed they do more than, to some extent, control the symptoms.

The most remarkable, as well as the most valuable, effect of the administration of aconite, is due to its power of *aborting* inflammatory action, if prescribed sufficiently early. I say the most valuable, because, although it is a great matter to be able to control inflammation, it is of much greater importance to be able to arrest or prevent it; and this power we can claim for aconite.

It is not my intention, in these few remarks, to enter into an examination of the physiological action of the drug, or to describe the mode of its action in disease, but merely to call attention to its remarkable efficacy in certain affections, and to refer briefly to some of the cases in which I have found it of signal service. My remarks are written purely for a practical purpose, and for the benefit of the busy practitioner.

The first disease to which I shall direct your attention, in which I have seen illustrated the abortive power of aconite, is pneumonia, and if it were the only affection which aconite could not only control, but also cut short its gravity is such that we should be warranted in giving the drug a prominent place in our pharmacopœia. If administered within a day or two after the symptoms are apparent enough to render the diagnosis certain—but of course the earlier the better—it will arrest the inflammation and effect a cure in from one to three or four days, the beneficial effects being manifest from the very commencement of its administration. The pain frequently begins to subside from the first, the skin becomes moist, the breathing more natural, and the patient appreciably better and more comfortable after each dose. I have used it frequently both in children and adults, and have never seen it fail to produce most satisfactory results. The dose I have generally employed for an adult is 5m. (Fleming's Tincture) at first and 1 or 2m. every hour after, modifying the dose according to circumstances. If the patient be debilitated from any cause, it must be prescribed cautiously, as I have seen it cause considerable alarm by producing delirium, nor are the beneficial effects of the drug any more, if so much, seen when it acts too powerfully.

I have not had sufficient experience to speak positively of its action in bronchitis, but so far as I have seen, it does not appear to exercise the same control over it as over pneumonia. I have, however, found it useful when there is much feverishness, with fullness of the head and flushing of the face. One reason why it has not proved so useful in bronchitis, as in many other affections, very probably is that we have not so generally an opportunity of treating bronchitis at the commencement of the inflammatory stage.

In cynanche tonsillaris I have found it exceedingly useful, both as an abortive and as a controlling or modifying agent. If properly administered during the inflammatory stage it will seldom fail to cut the attack short, and, if given at the very beginning, to abort it. If duly administered it not only cuts short the present attack, but after a time it seems to reduce or remove the liability to quinsy in persons subject to periodical attacks of it. It would take a considerable deal of evidence to establish this last fact, but I have seen it sufficiently often to warrant my referring to it.

I have not used it in croup, but Ringer says that the good effects of it, in catarrhal form of that malady, are as conspicuous as in quinsy.

Its use in fevers, especially in those of an inflammatory character, has been found very advantageous. It reduces the temperature and produces a very soothing effect from its action on the skin. Whether it will act as an abortive in such cases, or not, if given sufficiently early, I have not yet ascertained, but have every reason to believe it would.

It has been strongly recommended in acute rheumatism, but I have not found the effects so satisfactory as those from the use of actæa racemosa.

There is no doubt of its efficacy in erysipelas, especially in that form which is occasionally consequent upon vaccination, which I have seen it cut short in a few hours.

There was a case lately quoted in the *Practitioner* where its administration in frequently-repeated doses aborted milk abcess in twenty-four hours, and, though I have not yet tested it, it is, by analogy, quite what I should expect.

We have no better illustration of the efficacy and rapidity of the action of aconite than in common cold, "cold all through one," or "cold in the bones," as it is variously popularly described, when one feels as if he had been put "through a thrashing mill." Ringer (I think) states that one or two drops taken at bed-time will enable a person in such a state to rise quite well in the morning; and certainly in the doses I have mentioned, it affords very speedy relief.

It relieves that disagreeable affection, ringing in the ears, in many cases after a dose or two, and is said also to remove earache.

In the acute stage of gonorrhœa, when there is much pain and uneasiness, it affords marked relief.

There is one precaution in the use of it, to which Ringer directs attention. It is, he says, contraindicated in inflammatory affections where the temperature of the body is not above natural, so that in doubtful, and indeed in all cases, it is advisable to use the thermometer before prescribing it.

It is recommended in many other affections besides those I have referred to. I have called attention to those chiefly in which, in my own experience, I have found it invaluable. For a fuller account of its action and effects I cannot do better than refer my readers to Ringer's excellent *Handbook of Therapeutics*, than which, and especially the last edition, there is no book in the language more useful to the general practitioner.—*Practitioner.*

